# Supplementary figures and images for: The influence of the age-period-cohort effects on male suicide in Brazil from 1980 to 2019
Source: PLoS One. 2023 Apr 13;18(4):e0284224. doi: 10.1371/journal.pone.0284224 (PMC10101429; doi:10.1371/journal.pone.0284224)

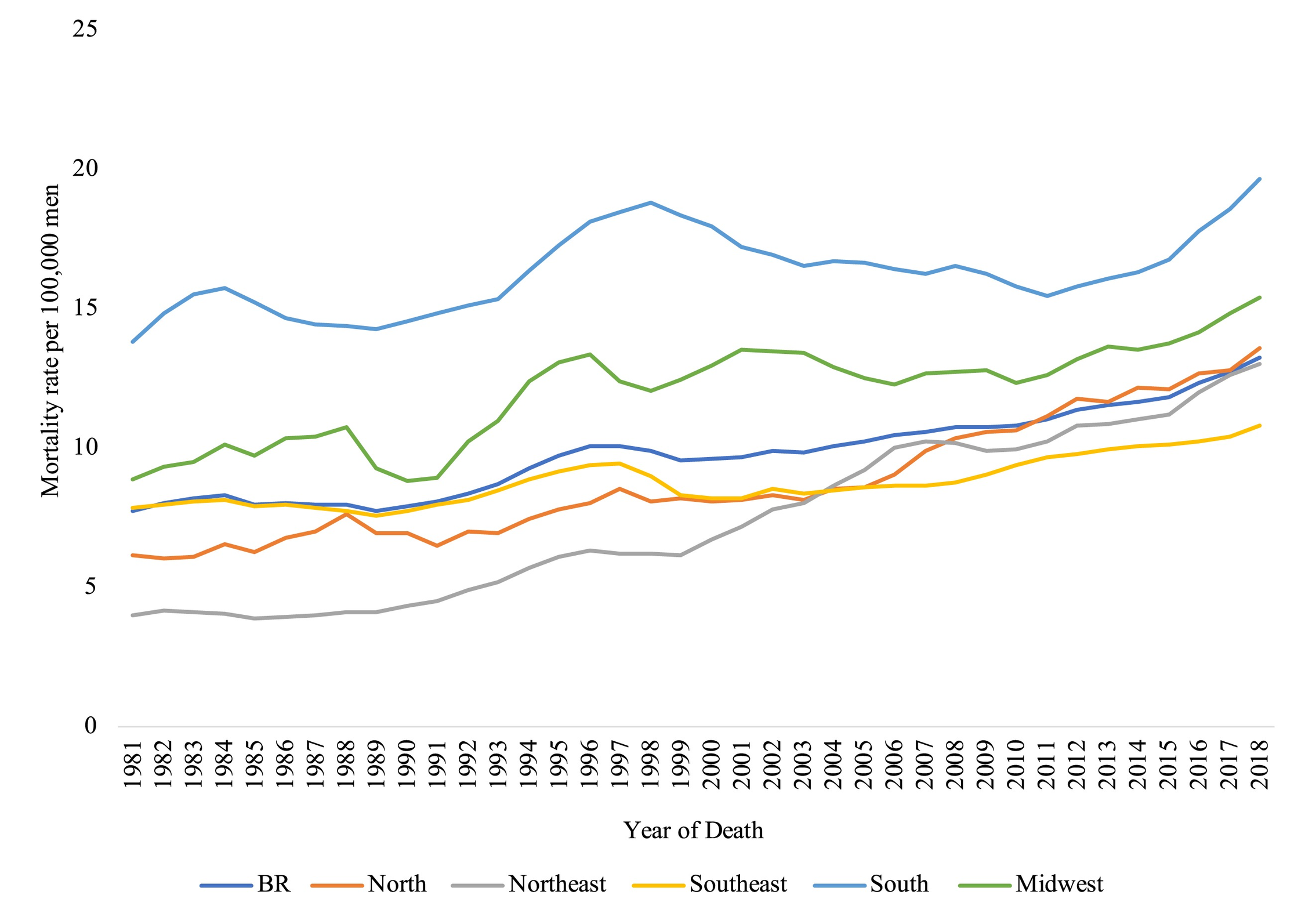

Supplement: S1 Fig — (TIF) [file pone.0284224.s001.tif]

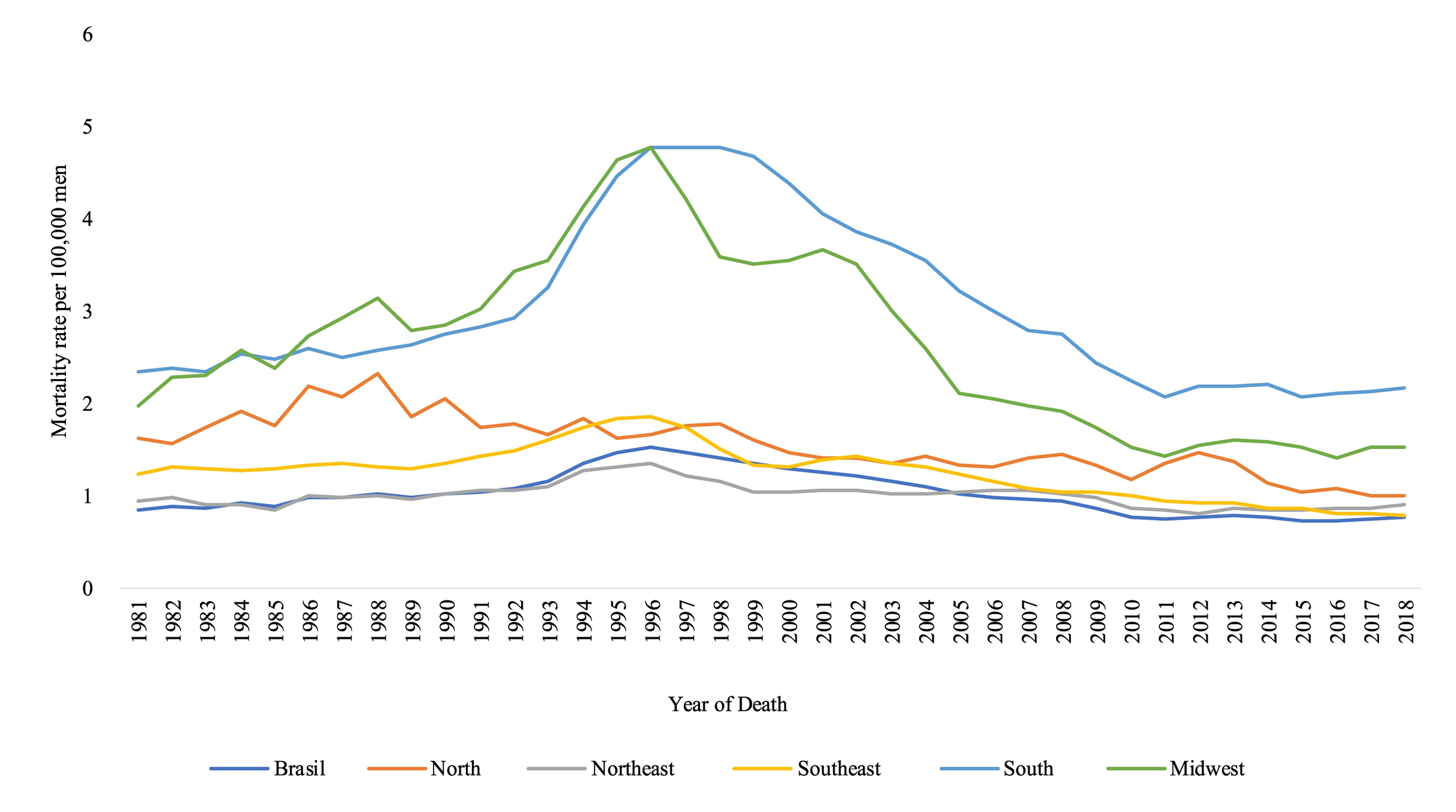

Supplement: S2 Fig — (TIF) [file pone.0284224.s002.tif]

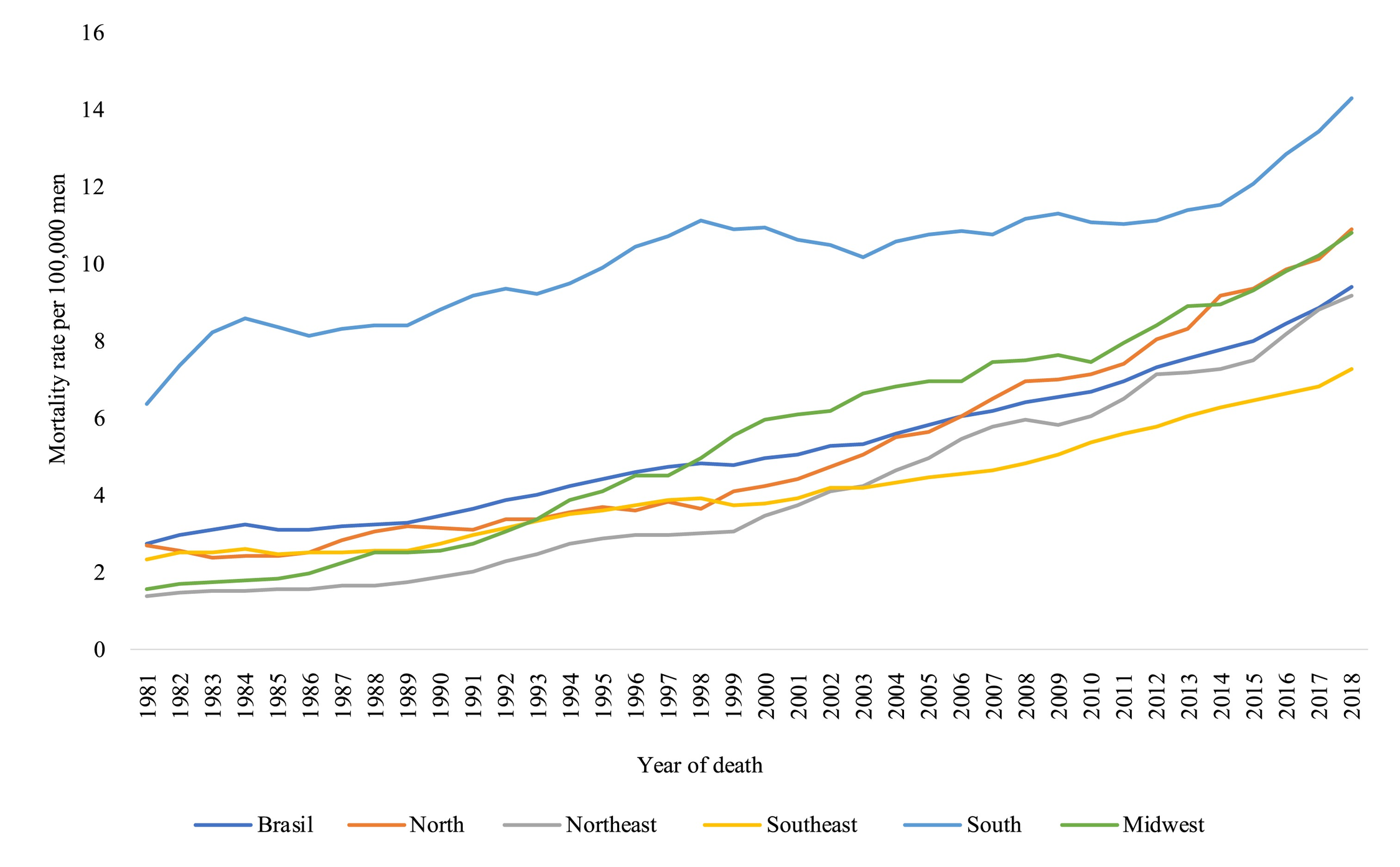

Supplement: S3 Fig — (TIF) [file pone.0284224.s003.tif]
