# Supplementary material for: The influence of the age-period-cohort effects on male suicide in Brazil from 1980 to 2019
Source: PLoS One. 2023 Apr 13;18(4):e0284224. doi: 10.1371/journal.pone.0284224 (PMC10101429; doi:10.1371/journal.pone.0284224)
Supplement: Attachment — Submitted filename: Response to reviewers.docx [file pone.0284224.s005.docx]

Response to reviewers1

*Dear Editor,*

*We would like to take this opportunity to thank the anonymous*

*reviewers that have taken part in this review process for their comments regarding the*

*manuscript.*

*All comments were relevant and incorporating the suggested changes has improved the manuscript. The new revised version of the the manuscript has followed the recommendations of Plos One Observation: The sentences in yellow have been removed or replaced by sentences in green. Some sentences were added in blue.*

Dear Authors,

Thank you for submitting your manuscript to PLOS ONE. After careful consideration, we feel that it has merit but does not fully meet PLOS ONE’s publication criteria as it currently stands. Therefore, we invite you to submit a revised version of the manuscript that addresses the points raised during the review process.

There are few weaknesses in the results that must be addressed in full before the article can be reconsidered for publication.

*We appreciate the valuable contributions, revised the entire results section, and made necessary corrections to the tables (header of some columns and table caption) , as shown below.*

Please submit your revised manuscript by 03/15/2023. If you will need more time than this to complete your revisions, please reply to this message or contact the journal office at [plosone@plos.org](mailto:plosone@plos.org). When you're ready to submit your revision, log on to <https://www.editorialmanager.com/pone/> and select the 'Submissions Needing Revision' folder to locate your manuscript file.

We look forward to receiving your revised manuscript.

Kind regards,

Fernanda Penido Matozinhos, Ph.D
Academic Editor
PLOS ONE

Journal Requirements:

Please review your reference list to ensure that it is complete and correct. If you have cited papers that have been retracted, please include the rationale for doing so in the manuscript text or remove these references and replace them with relevant current references. Any changes to the reference list should be mentioned in the rebuttal letter that accompanies your revised manuscript. If you need to cite a retracted article, indicate the article’s retracted status in the References list and also include a citation and full reference for the retraction notice.

*All articles used in the manuscript were reviewed, we included the doi or PMI of each of these, and none of the articles have been retracted.*
Additional Editor Comments (if provided):

Dear Editor and Author,

Thank you for the opportunity to review this manuscript. I am grateful for the invitation.

After careful consideration, I feel the manuscript explores a very important topic. The questions were responded and modifications in the text made the manuscript come to a satisfying result.
There are few weaknesses in the results that must be addressed in full before the article can be reconsidered for publication.

Kind regards,

*We appreciate the reviewer's note, all corrections have been included in the new version of the manuscript, as seen below.*
[Note: HTML markup is below. Please do not edit.]

Reviewers' comments:

Reviewer's Responses to Questions

**Comments to the Author**

1. If the authors have adequately addressed your comments raised in a previous round of review and you feel that this manuscript is now acceptable for publication, you may indicate that here to bypass the “Comments to the Author” section, enter your conflict of interest statement in the “Confidential to Editor” section, and submit your "Accept" recommendation.

Reviewer #3: All comments have been addressed

2. Is the manuscript technically sound, and do the data support the conclusions?

The manuscript must describe a technically sound piece of scientific research with data that supports the conclusions. Experiments must have been conducted rigorously, with appropriate controls, replication, and sample sizes. The conclusions must be drawn appropriately based on the data presented.

Reviewer #3: Yes

3. Has the statistical analysis been performed appropriately and rigorously?

Reviewer #3: Yes

4. Have the authors made all data underlying the findings in their manuscript fully available?

The [PLOS Data policy](http://www.plosone.org/static/policies.action#sharing) requires authors to make all data underlying the findings described in their manuscript fully available without restriction, with rare exception (please refer to the Data Availability Statement in the manuscript PDF file). The data should be provided as part of the manuscript or its supporting information, or deposited to a public repository. For example, in addition to summary statistics, the data points behind means, medians and variance measures should be available. If there are restrictions on publicly sharing data—e.g. participant privacy or use of data from a third party—those must be specified.

Reviewer #3: Yes

5. Is the manuscript presented in an intelligible fashion and written in standard English?

PLOS ONE does not copyedit accepted manuscripts, so the language in submitted articles must be clear, correct, and unambiguous. Any typographical or grammatical errors should be corrected at revision, so please note any specific errors here.

Reviewer #3: Yes

6. Review Comments to the Author

Please use the space provided to explain your answers to the questions above. You may also include additional comments for the author, including concerns about dual publication, research ethics, or publication ethics. (Please upload your review as an attachment if it exceeds 20,000 characters)

- Reviewer #3: The manuscript presented the effects of age, period, and cohort (APC) on total and firearm-related suicides in men in Brazil and its major regions from 1980 to 2019. It’s a well-written text and detailed document, mainly the methods. I need to highlight the importance of analyzing this information for creating and implementing public policies about health in Brazil. I agree with the changes made by the authors, guided by the reviewers, and I suggest only minor points that need revision.
  **Minor points**
- Table 2: correct the caption and put the abbreviations that you use in the table.

*We carry out the requested request, replace the acronym MRC with TC , which is in the table.*

***Change in the tables***

**Table 2. Standardized mortality rates per 100,000 men Brazil and its major regions, in five-year periods, in the period 1980-2019**

| **Locality** |  |  | **Suicide** |  |  |  |  |  |  |
| --- | --- | --- | --- | --- | --- | --- | --- | --- | --- |
|  | **1980 to 84** | **1985 to 89** | **1990 to 94** | **1995 to 99** | **2000 to 04** | **2005 to 09** | **2010 to 14** | **2015 to 2019** | **SAR^a^** |
| Brazil |  |  |  |  |  |  |  |  |  |
| UMR^b^ | 7.35 | 7.20 | 7.96 | 9.30 | 8.86 | 9.34 | 9.56 | 10.78 | 9.05 |
| TC^c^ | 8.98 | 8.76 | 9.14 | 10.68 | 10.11 | 10.03 | 11.05 | 12.32 | 10.22 |
| North |  |  |  |  |  |  |  |  |  |
| UMR^b^ | 4.06 | 4.62 | 5.19 | 5.91 | 6.33 | 7.30 | 8.05 | 9.70 | 7.01 |
| TC^c^ | 6.92 | 7.74 | 7.17 | 9.34 | 8.68 | 9.89 | 11.20 | 13.07 | 10.03 |
| Northeast |  |  |  |  |  |  |  |  |  |
| UMR^b^ | 3.23 | 3.21 | 4.36 | 6.22 | 6.35 | 8.12 | 8.38 | 9.79 | 6.74 |
| TC^c^ | 4.67 | 4.58 | 5.58 | 7.03 | 8.11 | 10.37 | 10.63 | 12.29 | 8.62 |
| Southeast |  |  |  |  |  |  |  |  |  |
| UMR^b^ | 7.44 | 7.03 | 7.61 | 8.37 | 7.54 | 7.81 | 8.47 | 8.47 | 8.02 |
| TC^c^ | 8.66 | 8.22 | 8.59 | 9.38 | 8.35 | 8.60 | 9.39 | 9.92 | 8.94 |
| South |  |  |  |  |  |  |  |  |  |
| UMR^b^ | 14.99 | 14.91 | 15.38 | 18.60 | 16.51 | 15.75 | 14.46 | 16.58 | 15.83 |
| TC^c^ | 17.08 | 16.52 | 16.58 | 19.91 | 17.45 | 16.50 | 15.20 | 17.49 | 16.93 |
| Midwest |  |  |  |  |  |  |  |  |  |
| UMR^b^ | 6.51 | 7.55 | 9.22 | 13.52 | 12.05 | 11.26 | 11.16 | 12.54 | 10.81 |
| TC^c^ | 9.97 | 11.16 | 10.78 | 13.90 | 13.77 | 12.81 | 12.90 | 14.44 | 12.83 |

^a^SAR= Standardized Average Rate;^b^UMR= Uncorrected mortality Rate; ^c^TC (total correction )= Mortality rates corrected for information quality and under-enumeration of deaths.

**Table 3. Deviance an p-value analysis in sequential construction of age, period, and cohort models for total suicides in men, according to Brazil its major regions, from 1980 to 2019.**

| Locality | | | |
| --- | --- | --- | --- |
|  | Brazil |  |  |
| Models | Df^a^ | Deviance Residual | p (>\|Chi\|) |
| Age | 115 | 15370.75 | |
| Age-drift | 114 | 12799.09 | <0.00001 |
| Age-Cohort | 111 | 12351.44 | <0.00001 |
| Age-Period- Cohort | 108 | 11690.76 | <0.00001 |
| Age-Period | 111 | 12030.81 | <0.00001 |
| Age-drift | 114 | 12799.09 | <0.00001 |
|  | North |  |  |
| Modelos | Df^a^ | Deviance Residual | p (>\|Chi\|) |
| Age | 115 | 1748.73 |  |
| Age-drift | 114 | 899.48 | <0.00001 |
| Age-Cohort | 111 | 774.40 | <0.00001 |
| Age-Period- Cohort | 108 | 764.44 | <0.00001 |
| Age-Period | 111 | 853.68 | <0.00001 |
| Age-drift | 114 | 899.48 | <0.00001 |
|  | Northeast |  |  |
| Age | Df^a^ | Deviance Residual | p (>\|Chi\|) |
| Age-drift | 115 | 8282.75 |  |
| Age-Cohort | 114 | 2459.19 | <0.00001 |
| Age-Period- Cohort | 111 | 2418.88 | <0.00001 |
| Age-Period | 108 | 2274.89 | <0.00001 |
| Age-drift | 111 | 2313.61 | <0.00001 |
| Age-drift | 114 | 2459.19 | <0.00001 |
|  | Southeast |  |  |
| Age | Df^a^ | Deviance Residual | p (>\|Chi\|) |
| Age-drift | 115 | 5189.92 |  |
| Age-Cohort | 114 | 4945.41 | <0.00001 |
| Age-Period- Cohort | 111 | 4623.20 | <0.00001 |
| Age-Period | 108 | 4508.44 | <0.00001 |
| Age-drift | 111 | 4829.88 | <0.00001 |
| Age-drift | 114 | 4945.41 | <0.00001 |
|  | South |  |  |
| Age | Df^a^ | Deviance Residual | p (>\|Chi\|) |
| Age-drift | 115 | 3879.20 |  |
| Age-Cohort | 114 | 3875.95 | <0.00001 |
| Age-Period- Cohort | 111 | 3764.64 | <0.00001 |
| Age-Period | 108 | 3530.84 | <0.00001 |
| Age-drift | 111 | 3662.38 | <0.00001 |
| Age-drift | 114 | 3875.95 | <0.00001 |
|  | Midwest |  |  |
| Age | Df^a^ | Deviance Residual | p (>\|Chi\|) |
| Age-drift | 115 | 1313.37 |  |
| Age-Cohort | 114 | 1146.13 | <0.00001 |
| Age-Period- Cohort | 111 | 1127.40 | <0.00001 |
| Age-Period | 108 | 1067.17 | <0.00001 |
| Age-drift | 111 | 1095.81 | <0.00001 |
| Age-drift | 114 | 1146.13 | <0.00001 |

^a^Degrees of freedom.

**Table 4. Deviance an p-value analysis in sequential construction of age, period, and cohort models for suicides by firearms in men, according to Brazil its major regions , from 1980 to 2019.**

| Locality | | | |
| --- | --- | --- | --- |
|  | Brazil |  |  |
| Age | Df^a^ | Deviance Residual | p (>\|Chi\|) |
| Age-drift | 115 | 5610.93 | |
| Age-Cohort | 114 | 4444.92 | <0.00001 |
| Age-Period- Cohort | 111 | 3863.52 | <0.00001 |
| Age-Period | 108 | 2768.71 | <0.00001 |
| Age-drift | 111 | 3014.51 | <0.00001 |
| Age-drift | 114 | 4444.92 | <0.00001 |
|  | North |  |  |
| Age | Df^a^ | Deviance Residual | p (>\|Chi\|) |
| Age-drift | 115 | 381.718 |  |
| Age-Cohort | 114 | 237.976 | <0.00001 |
| Age-Period- Cohort | 111 | 229.197 | <0.00001 |
| Age-Period | 108 | 223.507 | <0.00001 |
| Age-drift | 111 | 229.542 | <0.00001 |
| Age-drift | 114 | 237.976 | <0.00001 |
|  | Northeast |  |  |
| Age | Df^a^ | Deviance Residual | p (>\|Chi\|) |
| Age-drift | 115 | 557.75 |  |
| Age-Cohort | 114 | 493.79 | <0.00001 |
| Age-Period- Cohort | 111 | 444.34 | <0.00001 |
| Age-Period | 108 | 388.47 | <0.00001 |
| Age-drift | 111 | 410.41 | <0.00001 |
| Age-drift | 114 | 493.79 | <0.00001 |
|  | Southeast |  |  |
| Age | Df^a^ | Deviance Residual | p (>\|Chi\|) |
| Age-drift | 115 | 1989.28 |  |
| Age-Cohort | 114 | 1402.87 | <0.00001 |
| Age-Period- Cohort | 111 | 1182.73 | <0.00001 |
| Age-Period | 108 | 912.06 | <0.00001 |
| Age-drift | 111 | 1031.6 | <0.00001 |
| Age-drift | 114 | 1402.87 | <0.00001 |
|  | South |  |  |
| Age | Df^a^ | Deviance Residual | p (>\|Chi\|) |
| Age-drift | 115 | 2143.33 |  |
| Age-Cohort | 114 | 1928.99 | <0.00001 |
| Age-Period- Cohort | 111 | 1725.97 | <0.00001 |
| Age-Period | 108 | 995.56 | <0.00001 |
| Age-drift | 111 | 1073.80 | <0.00001 |
| Age-drift | 114 | 1928.99 | <0.00001 |
|  | Midwest |  |  |
| Age | Df^a^ | Deviance Residual | p (>\|Chi\|) |
| Age-drift | 115 | 1151.16 |  |
| Age-Cohort | 114 | 809.65 | <0.00001 |
| Age-Period- Cohort | 111 | 686.71 | <0.00001 |
| Age-Period | 108 | 385.03 | <0.00001 |
| Age-drift | 111 | 431.06 | <0.00001 |
| Age-drift | 114 | 809.65 | <0.00001 |

^a^Degrees of freedom.

**Tabela 5. Linear age-drift trend for Brazil and its geographic regions after fitting the APC model by estimable functions**

| Locality | | | |
| --- | --- | --- | --- |
| Brazil | Age -drift | CI-95% | Trend |
| Total suicide | 1.008 | 1.007-1.009 | Upward |
| Suicide by firearm | 0.984 | 0.983-0.985 | Downward |
| North | Age-drift | CI-95% | Trend |
| Total suicide | 1.02 | 1.019-1.021 | Upward |
| Suicide by firearm | 0.979 | 0.976-0.983 | Downward |
| Northeast | Age-drift | CI-95% | Trend |
| Total suicide | 1.03 | 1.029-1.032 | Upward |
| Suicide by firearm | 0.991 | 0.989-0.993 | Downward |
| Southeast | Age-drift | CI-95% | Trend |
| Total suicide | 1.004 | 1.004-1.005 | Upward |
| Suicide by firearm | 0.981 | 0.980-0.983 | Downward |
| South | Age-drift | CI-95% | Trend |
| Total suicide | 0.999 | 0.998-1.000 | Stationary |
| Suicide by firearm | 0.998 | 0.986-0.989 | Downward |
| Midwest | Age -drift | CI-95% | Trend |
| Total suicide | 1.007 | 1.006-1.009 | Upward |
| Suicide by firearm | 0.975 | 0.973-0.978 | Downward |

- I miss a comment at the end of the discussion about the period of the Covid-19 pandemic. You mention the need for future research, so I think it's interesting to add it, mainly because many of the causes of suicide were massive present during the pandemic period.

*We have included the suggested paragraph at the end of the discussion, before presenting the limitations of the study. As described below.*

***Change in the text***

These results point to the possibility of replacing suicide methods. This hypothesis should be studied in future studies, using interrupted time series or quasi-experimental studies using analysis or differences-in-differences, including socioeconomic and demographic variables. Furthermore, studies are needed to assess the impact of the COVID-19 pandemic on suicide mortality in men according to temporal factors (age, period, and cohort) and methods used. An increase in suicide rates is expected due to social isolation, fear of the unknown, fear of contaminating oneself or family members, socioeconomic impacts, such as unemployment, food insecurity, and lack of medical assistance to manage psychosocial problems [70,80 -81].
